# Prevalence of Depression among Rural Residents with Diabetes Mellitus: A Cross-Sectional Study from Northeast China

**DOI:** 10.3390/ijerph13060542

**Published:** 2016-05-28

**Authors:** Shasha Yu, Hongmei Yang, Xiaofan Guo, Liqiang Zheng, Yingxian Sun

**Affiliations:** 1 Department of Cardiology, The First Hospital of China Medical University, 155 Nanjing North Street, Heping District, Shenyang 110001, China; yidasasa047717@aliyun.com (S.Y.); yanghongmei047717@126.com (H.Y.); guoxiaofan047717@126.com (X.G.); 2 Department of Clinical Epidemiology, Shenjing Hospital of China Medical University, Shenyang 110001, China; zhengliqiang047717@126.com

**Keywords:** depressive symptoms, prevalence, rural, risk factors

## Abstract

Recent economic development in China has resulted in large increases in psychogenic and metabolic diseases. However, few studies have focused on the mental status of rural residents with diabetes. We aimed to investigate the prevalence of depressive symptoms among patients with diabetes to establish the association between depressive symptoms and socio-demographic and clinical factors. We conducted a cross-sectional analysis of 1187 patients with diabetes aged ≥35 years from rural Northeast China. Metabolic and anthropometric indicators were measured according to standard methods. Depressive symptoms were defined using the Patient Health Questionnaire-9 (PHQ-9). Five hundred and twenty-six residents (44.3%) of the total sample were male and 931 (78.4%) were <65 years old. One hundred and eight residents (8.76%) score ≥10 on the PHQ-9 scale. A statistically significant relationship was found between depressive symptoms and female gender, older age (≥65 years), high school or above education level, moderate physical activity, high family income, multiple additional illnesses, current alcohol consumption, and 7–8 h/d sleep duration. Multivariate analysis showed that female gender [odds ratio (OR) = 1.984, *p* = 0.028], high family income (OR = 0.483 for 5000–20,000 CNY/year, *p* = 0.011; OR = 0.356 for >2000 CNY/year, *p* = 0.003), 7–8 h/d sleep duration (OR = 0.453, *p* = 0.020), and having multiple additional illness (OR = 3.080, *p* < 0.001) were significantly associated with depressive symptoms. Prevalence of depressive symptoms in our study was high. Female gender and multiple illnesses were risk factors for depression, while long sleep duration and high family income seem to protect against depression among rural residents with diabetes in China.

## 1. Introduction

Over the past few decades, prediabetes and diabetes have become more prevalent in both developed and developing areas in China. The most recent nationwide study enrolled a representative sample of 46,239 adults from 14 provinces in China, and reported that the age-standardized prevalences of total diabetes and prediabetes were 9.7% and 15.5%, respectively [1]. Furthermore, this study claimed that the prevalence of diabetes was higher among urban than rural residents (11.4% *vs.* 8.2%) [1]. Yang and colleagues showed that the prevalence for diabetes and prediabetes in the rural population from Shandong province was 3.5% and 6.0%, respectively [2]. However, in a previous study, we found that the prevalence of diabetes in rural Northeast China was 10.6%, which was higher than in other rural areas and even close to that in urban areas [3].

Like diabetes, depression is also a major international public health problem with an increasing prevalence in China. Two national surveys of mental disorders, conducted in 1982 and 1993, revealed low, but increasing, lifetime prevalence (0.46% and 0.83%) of affective disorders in China [4,5]. A cross-sectional study of the burden of major depressive disorder among adults in urban China found a prevalence of 6.0% [6]. In contrast to urban areas, the prevalence of depressive symptoms among rural northeast China was 5.9%.

Both diabetes and depression are prevalent in developed countries and affect quality of life. It has been confirmed that diabetes partially increases the risk of depressive symptoms, while coexisting depression is associated with poor metabolic control and high risk of complications [7]. This results in higher healthcare costs and a heavy medical burden [8]. Therefore, it is necessary to estimate the possible association between diabetes and depression, especially to establish the possible risk factors for early intervention. The prevalence of depression varies widely due to variation in diabetes, race/ethnicity, and among developed and developing areas [9,10,11]. The relationship between depression and diabetes has been extensively studied in developed countries but few studies have focused on developing countries, and especially rural areas. In the present study, we aimed to determine the prevalence of depression in residents in rural Northeast China with diabetes and to clarify which factors might affect the prevalence of depression.

## 2. Methods

### 2.1. Study Population

This study is conducted in Liaoning Province which is located in Northeast China. A representative sample from the residents aged over 35 years was selected to describe the prevalence, incidence and natural history of cardiovascular risk factors in rural areas of Liaoning Province. We adopted a multi-stage, stratified random cluster-sampling scheme. In the first stage, three counties (Dawa, Zhangwu and Liaoyang) were selected from the eastern, southern, and northern region of Liaoning Province. In the second stage, one town was randomly selected from each county (a total of three towns). In the third stage, 8–10 rural villages from each town were randomly selected (a total of 26 rural villages). All the eligible permanent residents’ age ≥35 years from each village were invited to participate in the study. In total, there are 14,016 satisfactory participants enrolled in this study. Among them, 11,956 participants finished this study and the response rate was 85.3%. Participants who were pregnant, or had a malignant tumor or mental disorder were excluded. The study was approved by the Ethics Committee of China Medical University (Shenyang, China, AF-SDP-07-1, 0-01). All procedures were performed in accordance with the ethical standards. Written consent was obtained from each participant, after they had been informed of the objectives, benefits, medical items and confidentiality agreement of personal information. If the participants were illiterate, we obtained written informed consents from their close relatives. In this report, we focused on the residents with diabetes who lived in rural Northeast China, making a final sample size of 1187 (526 men and 661 women).

### 2.2. Data Collection and Measurements

Cardiologists and trained nurses collected the data during a single clinical visit using a standard questionnaire by face-to-face interview. Before the survey was performed, all eligible investigators were invited to attend organized training which included the purpose of this study, ways to administer the questionnaire, standard method of measurement, importance of standardization, and study procedures. All eligible investigators were asked to take a strict test after this training; only those who scored perfectly on the test could become investigators. During data collection, our inspectors had further instructions and support.

We used a standardized questionnaire to get data on demographic characteristics, lifestyle risk factors, dietary habits, family income, history of cardiovascular disease, and evaluation of psychological status in the face to face interview. There was a central steering committee with a subcommittee for quality control. Educational level was divided into primary school or below, middle school, and high school or above. Family income was classified as ≤5000, 5000–20,000 and >20,000 CNY/year. Self-reported sleep duration (including nocturnal and nap duration) was obtained from the questionnaire. The responses were categorized into four groups: ≤7, 7–8, 8–9 and >9 h/d.

Weight and height were measured to the nearest 0.1 kg and 0.1 cm, respectively, with the participants in lightweight clothing and without shoes. Waist circumference was measured at the umbilicus using a non-elastic tape (to the nearest 0.1 cm), with the participants standing at the end of normal expiration. Body mass index (BMI) was calculated as weight in kilograms divided by the square of the height in meters. Fasting blood samples were collected in the morning after ≥12 h fasting for all participants. Blood samples were obtained from an antecubital vein in Vacutainer tubes containing EDTA. Fasting plasma glucose (FPG), total cholesterol (TC), low-density lipoprotein cholesterol (LDL-C), high-density lipoprotein cholesterol (HDL-C), triglyceride (TG) and other routine blood biochemical indexes were analyzed enzymatically on an autoanalyzer. All laboratory equipment was calibrated and blinded duplicate samples were used.

### 2.3. Definitions

Diabetes was diagnosed according to the WHO criteria [12]: fasting plasma glucose ≥ 7 mmol/L (126 mg/dL) and/or being on treatment for diabetes. Two definitions for impaired FPG were used. According to the 1997 American Diabetes Association (ADA) criteria, impaired FPG was defined as 6.1–6.9 mmol/L (110–126 mg/dL), and according to the ADA 2010 criteria, impaired FPG was defined as fasting plasma glucose 5.6–6.9 mmol/L (100–126 mg/dL) [13].

Depressive symptoms were assessed with PHQ-9, which is widely used in primary health centers for screening of depression [14,15]. Each of the nine PHQ depression items corresponds to one of the DSM-IV diagnostic criterion for symptoms for major depressive disorder [16]. The subjects were asked how often, over the past 2 weeks, they had been bothered by each of the depressive symptoms. The response options were “not at all”, “several days”, “more than half the days”, and “nearly every day” and were scored as 0, 1, 2, and 3, respectively. PHQ-9 scores ranged from 0 to 27, with scores of ≥5, ≥10, and ≥15, representing mild, moderate, and severe levels of depression, respectively [17]. Individuals with a PHQ-9 score >10 were considered to have severe depression symptoms [18].

Physical activity includes occupational and leisure-time physical activity [19]. The subjects reported their occupational physical activity according to the following three categories: (i) light, which indicated easy physical activity, sedentary work (e.g., secretary); (ii) moderate for work including standing and walking (e.g., store assistant); and (iii) active, which indicated work including walking and lifting, or heavy manual labor (e.g., industrial or farm work). Self-reported leisure physical activity was classified into three categories: (i) low, light levels of occupational and leisure-time physical activity; (ii) moderate, moderate or high levels of occupational or leisure-time physical activity; and (iii) high, moderate or high levels of both occupational and leisure-time physical activity.

### 2.4. Statistical Analysis

Descriptive statistics were calculated for all the variables, including continuous variables (reported as mean values and standard deviations) and categorical variables (reported as numbers and percentages). Differences between different depressive status groups were evaluated using Student’s *t*-test, ANOVA, non-parametric test or the χ^2^ test as appropriate. Multivariate logistic regression analyses were used to identify the possible risk factors of depressive symptoms with odds ratios (ORs) and corresponding 95% confidence intervals (CIs) calculated. All the statistical analyses were performed using SPSS version 17.0 software (SPSS Inc., Chicago, IL, USA) and *p* < 0.05 were considered statistically significant.

## 3. Result

Table 1 shows that the majority (931; 78.4%) of participants were younger than 65 years old. Compared with those aged 35–45 years, older participants had a higher risk of depressive symptoms, although there was only a significant difference among those aged >65 years (OR = 2.233 for 45–54 years, *p* = 0.106; OR = 2.146 for 55–64 years, *p* = 0.117; OR = 3.611 for >65 years, *p* = 0.009). The majority (661; 55.7%) of participants were female. Women seemed to have a higher risk of depressive symptoms than men (OR = 2.880, *p* < 0.001). The largest proportion (696; 58.6%) of participants had been to primary school or below, while only a few had finished high school or above (99; 8.3%). The marital status was married (*n* = 1068; 90.0%) or single/divorced/widowed (*n* = 119, 10.0%). The majority of participants were non-smokers (*n* = 821, 69.2%) and non-drinkers (*n* = 930, 78.3%) at the time of the study. Only 6.1% of participants undertook vigorous physical activity. Moderate physical activity was a protective factor for depressive symptoms. Higher annual income was associated with lower risk of depressive symptoms (OR = 0.465 for 5000–20,000 CNY/year, *p* = 0.002; OR = 0.292 for >20,000 CNY/year, *p* < 0.001).

**Figure 1:**
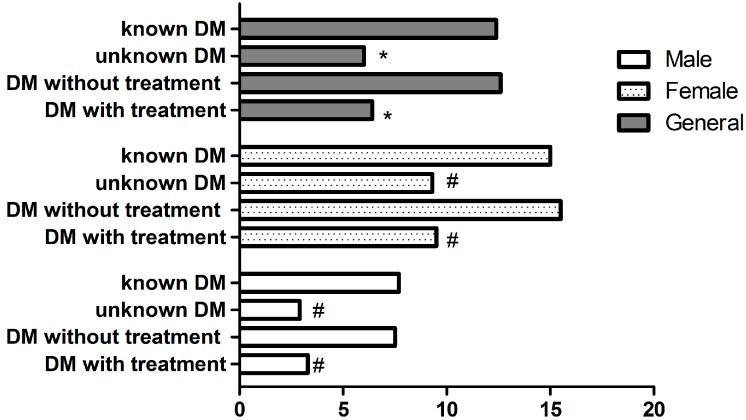
Prevalence of depressive symptoms among those residents known or unknown diabetes and control or uncontrol diabetes. DM: diabetes. * means *p* < 0.001; # means *p* < 0.05.

Most of the residents had ≤ 7 h/d sleep duration and longer sleep duration seemed to decrease the risk of depressive symptoms (OR = 0.395 for 7–8 h/d, *p* = 0.002; OR = 0.683 for 8–9 h/d, *p* = 0.229; OR = 0.876 for >9 h/d, *p* = 0.711). A number of residents (22.4%) had more than two additional illnesses. However, more than two additional illnesses was related with higher risk of depressive symptoms (OR = 3.899, *p* < 0.001). Figure 1 shows that the prevalence of depressive symptoms was 7.5% and 3.3% among control and uncontrolled men (*p* = 0.032), while among women it was 15.5% and 9.5%, respectively (*p* = 0.013). Men who knew that they had diabetes had a higher prevalence than those who did not know (7.7% *vs.* 2.9%, *p* = 0.013), which was similar to women (15.0% *vs.* 9.3%, *p* = 0.016).

**Figure 2:**
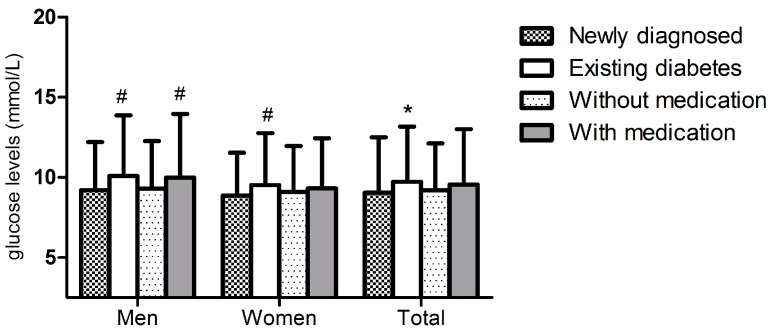
The average glucose levels of residents with newly diagnosed versus those existing diabetes and of those without and with medication. # means *p* < 0.05. * means *p* < 0.001.

Figure 2 shows the average glucose levels of those with newly diagnosed *versus* existing diabetes and of those with and without medication by gender. The mean value of glucose among those newly diagnosed with diabetes was lower than in those with existing diabetes among both men and women (9.20 ± 3.0 *vs.* 10.09 ± 3.78, *p* = 0.004; 8.87 ± 2.67 *vs.* 9.52 ± 3.25, *p* = 0.005). Besides, residents with medication had significantly higher mean glucose level than those without medication among men but not among women (9.99 ± 3.96 *vs.* 9.29 ± 2.97, *p* = 0025; 9.32 ± 3.13 *vs.* 9.09 ± 2.87, *p* = 0.334).

Multivariate analysis (Table 2) showed that: (1) patients with diabetes with higher annual income were less likely to have depression (OR = 0.483 for 5000–20,000 CNY/year, *p* = 0.011; OR = 0.356 for >20,000 CNY/year, *p* = 0.003) than those with lower annual income; (2) female patients with diabetes were more likely to have depression (OR = 1.984, *p* = 0.028) than male patients; (3) patients with diabetes and multiple additional illnesses were more likely to have depression (OR = 3.080, *p* < 0.001) than those with only one additional illness; and 4) patients with diabetes with longer sleep duration were less likely to have depression (OR = 0.453 for 7–8 h/d, *p* = 0.020) than those with <7 h/d sleep duration.

## 4. Discussion

This study investigated the prevalence of depressive symptoms among adult residents from rural Northeast China with diabetes and identified demographic and disease-related risk factors for depression. We showed that 8.8% of the screened patients had depressive symptoms while only 5.7% of the non-diabetic participants had depressive symptoms (*p* < 0.01). Most residents who were potential cases of depression were women, older (>65 years), and had multiple additional illness. Those who had high school or above educational status, moderate physical activity, higher annual income, longer sleep duration, and were current drinkers seemed to be less likely to have depressive symptoms. Multivariate analysis revealed that among the general population, female gender, higher annual income and longer sleep duration decreased the risk of depressive symptoms, while having multiple additional illnesses increased the risk.

Many previous studies that estimated the association between diabetes and depression concluded that depression was more prevalent among patients with diabetes. A meta-analysis of 51,331 people showed that the prevalence of depression was significantly higher in patients with diabetes (17.6% *vs.* 9.8%) [10]. In our study, we found that 8.8% of residents with diabetes had depressive symptoms, which was higher than in the general population (5.9%). Data from the 2006 Behavioral Risk Factor Surveillance System, a standardized telephone survey among US adults, showed that the age-adjusted prevalence rate of major depression was 8.3% [9]. The prevalence of depression among patients with diabetes varied according to regional divergence, from 9.8% in the Basque Country to 49.6% in Saudi Arabia and 40.3% in Nepal [20,21]. The lower prevalence in our study might have been partially due to the different diagnostic criteria and the enrolled participants. Some studies have reported that the higher rate of uncontrolled diabetes might partially account for the high prevalence of depression [22,23]. However, in our study, the rate of uncontrolled diabetes was lower than in those studies (62.0%) [11].

Studies that estimated the prevalence of depression among patients with diabetes found that gender, ethnicity, married status and poor economic conditions were associated with depression [9]. Women tend to have a higher prevalence of depressive symptoms among the general population and among patients with diabetes [9,24]. Al-Amer and colleagues claimed that women are more likely to develop depression than men (OR = 1.91, *p* = 0.001) [25]. In our study, women had a 1.984-fold higher risk of depression than men. El Mahalli revealed that unmarried patients with diabetes were three times more depressed than married ones [26]. In our study, unmarried residents had a higher prevalence of depression than married ones, although the difference was not significant (12.6% *vs.* 8.3%, *p* > 0.05). A study in the U.S. found that there were almost 25-fold differences in the rate of major depression among racial/ethnic subgroups [9]. As a result of the small number of patients from ethnic minorities in our study (5.4%), there was no difference among different ethnic groups. As for the association between income and depression, some studies concluded that higher monthly income is associated with increased severity of depression, while others have reported that major depression and depressive symptoms are associated with lower personal income [27,28]. Data from our study support the first conclusion—that higher income seems to be a protective factor for depressive symptoms among residents with diabetes in rural China. Studies that claimed wealthy people are more likely to suffer from depression suggested that a higher income would may result in poor health habits, e.g., poor nutrition, limited exercise, excessive caloric intake, and smoking and drinking [29]. In contrast, in another study, it was suggested that residents with lower income are more likely to be depressed and the burden of low income and disability could lead to depression [27]. Further longitudinal studies need to examine the causal relations. Chang and colleagues conducted a study in rural communities of Missouri, Tennessee and Arkansas, and showed that short sleep duration (<7 h/night) was positively associated with increased depressive symptoms [30]. In agreement with many other similar studies, our study confirmed that relatively longer sleep duration protects residents from depressive symptoms [31]. Furthermore, previous epidemiological investigations have indicated that there is a U-shaped relationship between sleep duration and depressive symptoms [32,33]. In our study, there was also a U-shaped relationship; however, it did not reach statistical significance. Many studies have confirmed the relationship between alcohol use and depression [34,35]. However, in our present study, the relationship between depressive symptoms and alcohol use did not reach statistical significance. The possible reasons are that the previous studies were mostly conducted among the general population or alcohol users [35,36]. There was a lack of data among patients with diabetes. Besides, in our study, those residents who were current drinkers had a lower rate of depressive symptoms than those who were not drinking. We assume that there might be somehow related with relived effect of mood by alcohol to those rural residents. Through drinking, they might somehow release their negative emotions. Second, the amount of alcohol that rural residents consume might be less than in westerners. A further study that considers the amount of alcohol consumed is needed.

## 5. Conclusions

Some limitations in the present study warrant mention. First, due to the cross-sectional design of our study, we cannot make inferences about causality. Second, we relied solely on the results of fasting plasma glucose tests to diagnose diabetes in the residents we enrolled in this study, despite the fact that HbA_1c_ has been integrated into the diagnostic criteria for diabetes in the updated 2010 ADA guidelines. This means that some diabetic subjects may have been left out. Third, this study used a questionnaire and did not use a comprehensive psychiatric evaluation. We did not confirm a definitive diagnosis of depression. Finally, in this study, we used the subjects’ own description of activity to define leisure self-activity, which may have introduced bias.

The prevalence of depressive symptoms among residents from rural China with diabetes is higher than that among the general population. Being a woman and having additional illnesses were significant predictors and were associated with increasing likelihood of developing major depressive disorders. In contrast, having higher annual income and relatively longer sleep duration seemed to protect residents with diabetes against depression. Regular psychological screening is recommended among residents in rural China with diabetes.

## Figures and Tables

**Table 1:** Unvaried analysis of factors associated with depressive symptoms.

Variables	Total *n* = 1187	PHQ-9 Depressive Score		Unadjusted OR (95% CI)	*p*-Value
		<10 *n* = 1083	≥10 *n* = 104		
**Age group (years)**					
35–44	127 (10.7)	*122 (96.1)*	*5 (3.9)*	Reference	
45–54	334 (28.1)	*306 (91.6)*	*28 (8.4)*	2.233 (0.843, 5.916)	0.106
55–64	470 (39.6)	*432 (91.9)*	*38 (8.1)*	2.146 (0.827, 5.571)	0.117
≥65	256 (21.6)	*223 (87.1)*	*33 (12.9)*	**3.611 (1.374, 9.488)**	**0.009**
**Gender**					
Male	526 (44.3)	**502 (95.4)**	**24 (4.6)**	Reference	
Female	661 (55.7)	**581 (87.9)**	**80 (12.1)**	**2.880 (1.797, 4.615)**	**<0.001**
**Educational status**					
Primary school or below	696 (58.6)	*625 (89.8)*	*71 (10.2)*	Reference	
Middle school	392 (33.0)	*362 (92.3)*	*30 (7.7)*	0.730 (0.467, 1.140)	0.166
High school or above	99 (8.3)	*96 (97.0)*	*3 (3.0)*	**0.275 (0.085, 0.891)**	**0.031**
**Ethnicity**					
Han	1123 (94.6)	1026 (91.4)	97 (8.6)	Reference	
Others ^a^	64 (5.4)	57 (89.1)	7 (10.9)	0.770 (0.342, 1.734)	0.528
**Marital status**					
Married	1068 (90.0)	979 (91.7)	89 (8.3)	Reference	
Others	119 (10.0)	104 (87.4)	15 (12.6)	1.587 (0.885, 2.843)	0.121
**Physical activity**					
Light	472 (39.8)	*417 (88.3)*	*55 (11.7)*	Reference	
Moderate	642 (54.1)	*598 (93.1)*	*44 (6.9)*	**0.558 (0.368,0.845)**	**0.006**
Severe	73 (6.1)	*68 (93.2)*	*5 (6.8)*	0.557 (0.215, 1.443)	0.228
**Annual income (CNY/year)**					
≤5000	167 (14.1)	**139** (83.2)	**28** (16.8)	Reference	
5000–20,000	642 (54.1)	**587** (91.4)	**55** (8.6)	**0.465 (0.285, 0.760)**	**0.002**
>20,000	378 (31.8)	**357** (94.4)	**21** (5.6)	**0.292 (0.160, 0.531)**	**<0.001**
**Sleep duration (h/d)**					
≤7	613 (51.6)	*546 (89.1)*	*67 (10.9)*	Reference	
7–8	303 (25.5)	*289 (95.4)*	*14 (4.6)*	**0.395 (0.218, 0.715)**	**0.002**
8–9	168 (14.2)	*155 (92.3)*	*13 (7.7)*	0.683 (0.368, 1.271)	0.229
>9	103 (8.7)	*93 (90.3)*	*10 (9.7)*	0.876 (0.435, 1.764)	0.711
**Current smoking status**					
No	821 (69.2)	*741 (90.3)*	*80 (9.7)*	Reference	
Yes	366 (30.8)	*342 (93.4)*	*24 (6.6)*	0.650 (0.405, 1.044)	0.075
**Current drinking status**					
No	930 (78.3)	**833** (89.6)	**97** (10.4)	Reference	
Yes	257 (21.7)	**250** (97.3)	**7** (2.7)	**0.240 (0.110, 0.525)**	**<0.001**
**Body mass index (kg/m^2^)**					
<25	451 (38.0)	403 (89.4)	48 (10.6)	Reference	
25–29	571 (48.1)	527 (92.3)	44 (7.7)	0.701 (0.456, 1.077)	0.214
>30	165 (13.9)	153 (92.7)	12 (7.3)	0.658 (0.341, 1.273)	0.854
**Number of additional illnesses**					
≤1	883 (77.6)	**837 (94.8)**	**46 (5.2)**	Reference	
≥2	255 (22.4)	**210 (82.4)**	**45 (17.6)**	**3.899 (2.516, 6.041)**	**<0.001**

Bold means *p* < 0.001; Italic means *p* < 0.05.

**Table 2:** Multivariate analysis of factors associated with depressive symptom among diabetic residents *.

Variables	Odds Ratio	95% CI	*p*-Value
**Gender**			
Male	1 (reference)		
Female	1.984	1.077, 3.656	0.028
**Annual income (CNY/year)**			
≤5000	1 (reference)		
5000–20,000	0.483	0.276, 0.847	0.011
>20,000	0.356	0.181, 0.700	0.003
**Sleep duration (h/d)**			
≤7	1 (reference)		
7–8	0.453	0.233, 0.881	0.020
8–9	0.883	0.451, 1.727	0.715
>9	1.315	0.592, 2.921	0.502
**Number of additional illness**			
≤1	1 (reference)		
≥2	3.080	1.886, 5.028	<0.001

* Adjusted for gender, age group, educational status, marital status, ethnicity, physical activity, annual income, sleep duration, current smoking and drinking status, body mass index, number of additional illness, aware or unaware diabetes and control or uncontrolled diabetes. Only data with significantly difference was shown in the table. Abbreviations: CI, confidence interval, β coefficient of predictor variables, BMI, Body Mass Index, OR, Odds Ratio.
